# Dataset of KCNQ1, KCNN4, KATP channel expression and dexamethasone modulation of protein kinase signaling in airway epithelial cells

**DOI:** 10.1016/j.dib.2019.104642

**Published:** 2019-10-22

**Authors:** Darina Hynes, Brian J. Harvey

**Affiliations:** a Department of Molecular Medicine, Royal College of Surgeons in Ireland, Beaumont Hospital, Dublin 9, Ireland; b Centro di Estudios Cientificos CECs, Valdivia, Chile

**Keywords:** Dexamethasone, Non-genomic, Airway epithelium, KCNQ1, KCNN4, KATP channels, Protein kinases

## Abstract

Dexamethasone produces anti-secretory responses in airway epithelium through the inhibition of basolateral membrane K^+^ channels [1–3]. We have used the human bronchial epithelial cell line 16HBE14o^−^ to investigate the effects of dexamethasone on the expression of K^+^ channels and regulatory protein kinases. The data demonstrate the expression of three distinct K^+^ channel types – KCNQ1:KCNE3, KCNN4 and KATP which are differentially regulated by protein kinase A and protein kinase C. The data also provide evidence for rapid non-genomic actions of dexamethasone on PKC and PKA phosphorylation and their association with the various K^+^ channel sub-types. Biotinylation experiments provide data on the effects of dexamethasone on membrane expression of the K^+^ channels. Antibody co-immunoprecipitation, rtPCR and western blotting data are given for the non-genomic dexamethasone transcription-cell signaling pathway involving G_i_-protein coupled receptor, PKC, adenylyl cyclase Type IV, cAMP, PKA and ERK1/2 activation.

**Table:** Specifications table

Subject	Endocrinology
Specific subject area	Dexamethasone regulation of airway epithelial K^+^ channels
Type of data	Tables Figures
How data were acquired	rtPCR, western blotting, antibody co-immunoprecipitation, biotinylation
Data format	Raw Analysed
Parameters for data collection	For the data reported here, 16HBE14o^−^ cells were plated in 75 cm^2^ polystyrene culture flasks and grown to confluence in Eagle's Minimum Essential Medium (EMEM) supplemented with 10% foetal bovine serum, 1% non-essential amino acids, 1% l-glutamine, 50 U/ml penicillin, 0.05 mg/ml streptomycin. Western blot analysis was carried out as standard. Protein was transferred to nitrocellulose membranes, blocked in 1× TBS with Tween (0.3%) (1× TBST) and 5% non-fat dry milk for 1 h. Membranes were incubated with the appropriate primary antibody overnight at 4 °C and incubated for 1 h at room temperature with the appropriate secondary antibody. Membranes were washed in 1× TBST 0.3% three times for 15 min. Bands were detected using autoradiographic film and chemiluminescence. Human KCNQ1 (hKCNQ1) cloned into pTLN6, was kindly provided by Prof. Thomas Jentsch (Leibniz-Institute for Molecular Pharmacology, Berlin, Germany). Cell surface labelling with biotin-LC-hydrazide: Confluent 16HBE14o^−^ cell monolayers were washed three times with ice cold PBS-CM (PBS supplemented with 1 mM MgCl_2_ and 0.1 mM CaCl_2_) and sodium periodate in PBS-CM added at a concentration of 10 mM for 30 minutes. After removal of the sodium periodate, the monolayers were washed twice with PBS-CM and three times with 0.1 M sodium acetate-CM (0.1 M sodium acetate (pH 5.5) supplemented with 1 mM MgCl_2_, 0.1 mM CaCl_2_). 0.1 mM biotin–LC–hydrazide (0.37 mg/ml made up in ice-cold Na acetate-CM, pH 5.5) was added to the cells for a further 30 minutes. Termination of the labelling was brought about by removal of the biotin with subsequent washing of the monolayers three times in PBS. Cells were solubilised by the addition of 0.5 ml/well lysate buffer and incubation at 4 °C for 1 hour on a rotating wheel. Insoluble material was pelleted by centrifugation for 15 minutes at 14,000 rpm and the supernatant removed to fresh microfuge tubes. Protein concentrations of samples were calculated by comparison to the BSA standards. Specific proteins were immunoprecipitated by incubating the extracts with 20 μl protein-G Sepharose beads. Cell surface biotin-labelled proteins were identified by incubation of the membrane with horseradish peroxidase (HRP)-conjugated streptavidin for 60 minutes at room temperature and visualisation detected by an enhanced chemiluminescent procedure (ECL-plus).
Description of data collection	Densitometric analysis of western blots and PKA, PKC assays were performed using Gene tools software (Syngene, Cambridge UK). Background was subtracted from each densitometric value. Statistical analysis of the data was performed using a *t*-test for comparison between two groups. One-way ANOVA and Tukeys post-hoc test was used for multiple group analyses. *P*-values of 0.05 and less were considered to be significant. Statistical operations were performed using Excel® software (Microsoft) and GraphPad® InStat version 3.5 (GraphPad Software, San Diego). Data are expressed as means ± SEM. Number of samples (*n*) refers to the number of times the independent experiments were performed.
Data source location	RCSI-ERC, Beaumont Hospital City/Town/Region: Dublin Country: Ireland Latitude and longitude: 53.3902738, -6.2209754
Data accessibility	With this article
Related research article	Author's name Darina Hynes and Brian J. Harvey Title **Dexamethasone reduces airway epithelial Cl**^**−**^**secretion by rapid non-genomic inhibition of KCNQ1, KCNN4 and KATP K**^**+**^**channels** Journal Steroids Special Issue RRSH2018 DOI

**Table:** 

**Value of the Data** • Data demonstrate non-genomic regulation of K^+^ channels and protein kinases by dexamethasone in airway epithelial cells. • Data provide insights into steroid regulation of epithelial ion channels via protein kinases. • Data provide insights into molecular mechanisms of non-genomic actions of glucocorticoids. • Data are useful in assessing clinical use of glucocorticoids to modulate hypersecretion in asthma and COPD

## 1 Data

We have previously described a rapid non-genomic anti-secretory effect of corticosteroids on airway Cl^-^ secretion via inhibition of basolateral membrane K^+^ channels [[1], [2], [3]]. Here we present the original data underpinning these findings.

## 2 Experimental design, materials, and methods

### 2.1 Expression of the KCNN4 protein in 16HBE14o^−^ airway epithelial cells

**Fig. 1:**
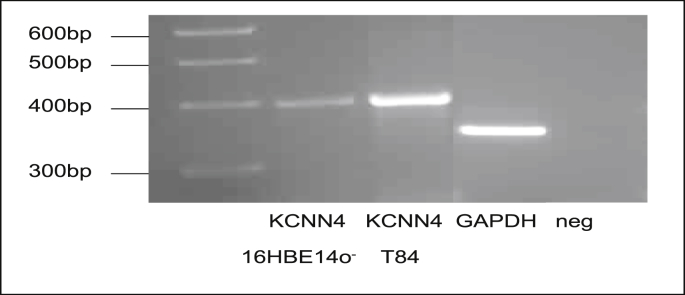
**Semi-quantitative RT-PCR analysis of KCNN4 expression in 16HBE14o**^**−**^**cells.** Total RNA was isolated from 16HBE14o^−^ cells using RNA easy kit (Qiagen). cDNA was reverse transcribed using poly dT primers and ImProm*™ Reverse Transcriptase System* (Promega, USA) and 1 μl of this reaction was directly amplified using GoTaq® Green Master Mix. (Promega, USA) using specific primers for human KCNN4 isoform and synthesised by MWG Biotech (Germany). The PCR reaction produced DNA fragments at the expected length for KCNN4 in T84 and 16HBE14o^−^ cells. GAPDH (cDNA and GAPDH primer pairs) was used as a control and neg (negative control, primers pairs without cDNA).

**Fig. 2:**
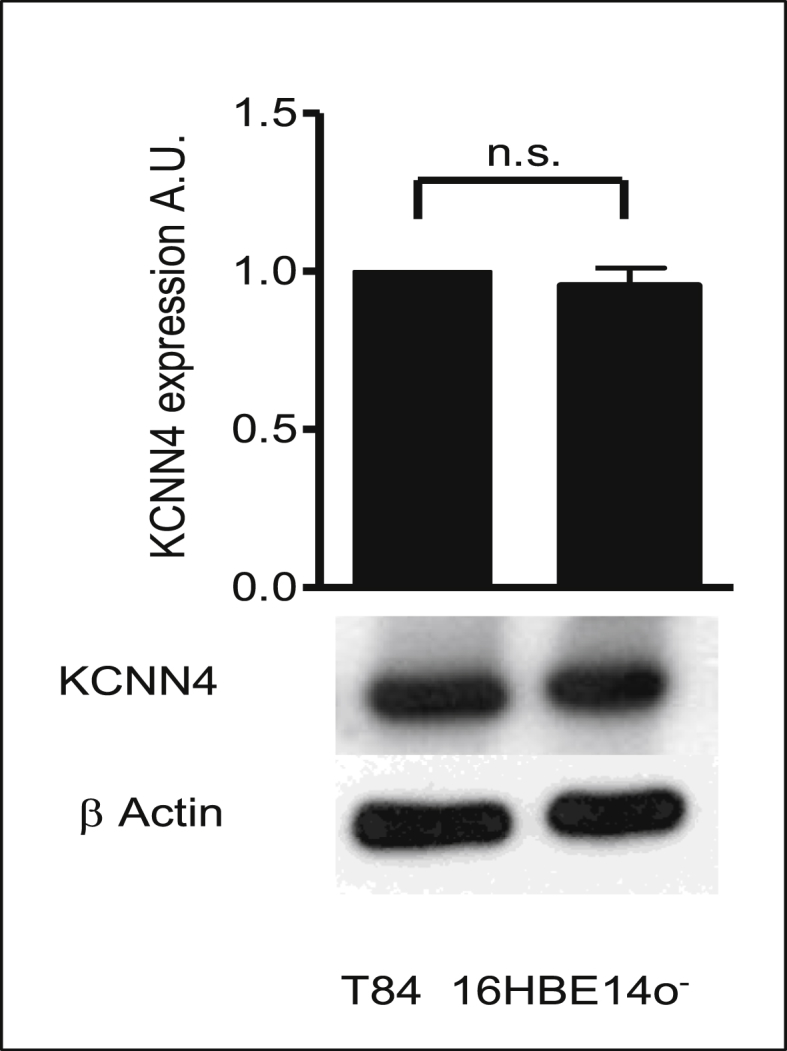
**KCNN4 protein expression in 16HBE14o**^**−**^**cells.** Western blot analysis of KCNN4 proteins in human bronchial epithelial cells. Total protein (100 μg/lane) was transferred to nitrocellulose membrane after fractionating by SDS-PAGE and blotted with anti-KCNN4. Bands at 46 kDa corresponding to KCNN4 were detected. β-actin was used as a control to estimate protein loading. Values represent mean ± SEM, n = 3; n.s. denotes values were not significant between T84 and 16HBE14o^−^ samples. Statistical analysis was performed using the Student's paired *t*-test.

Western blot analysis was performed to determine expression of the KCNN4 channel in 16HBE14o^−^ cells. Primers specific for KCNN4 (GenBank accessionNo. AF000972) were designed using the Primer 3 software (Table 1). A PCR product of the expected size was generated from complimentary DNA extracted from 16HBE14o^−^ cells (Fig. 1). The T84 cell line was used as a positive control. As illustrated in Fig. 2, an anti-KCNN4 antibody recognized prominent bands with the molecular masses of 46 kDa.

**Table 1:** First-strand cDNA was subjected to PCR amplification using primers designed with the Primer 3 PCR primer design program (Whitehead Institute for Biomedical Research).

Protein	Primer Sequence 5′ → 3′	Product size bp
KCNN4 (F)	GCCGTGCGTGCAGGATTTAGG	403
KCNN4 (R)	GCCCGGCACCACGTCACCATA	
GAPDH (F)	CATTGGGGGTAGGAACACGGA	373
GAPDH(R)	GCCAAAAGGGTCATCATCTCCG

### 2.2 Membrane expression of KCNN4 in 16HBE14o^−^ cells

**Fig. 3:**
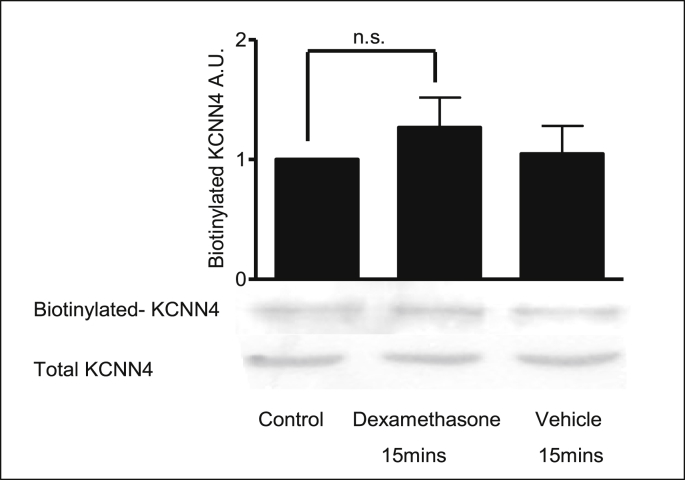
**KCNN4 channels are present at the membrane in 16HBE14o**^**−**^**cells.** Representative Western blot of biotin-labelled cell surface KCNN4 protein. Dexamethasone (1nM) or vehicle (methanol, 0.001% v/v) was added to 16HBE14o^−^ cells for 15 mins. Barchart summary for the effect of dexamethasone on cell surface biotinylation in 16HBE14o^−^ cells. Values are mean ± SEM, n = 3, n.s. denotes values are not significant. Statistical analysis was performed by one-way ANOVA followed by Tukey's multiple comparison tests.

Biotinylation experiments were performed to investigate the effect of dexamethasone on the expression and membrane localization of KCNN4 channels (Fig. 3). The presence of KCNN4 at the basolateral membrane was confirmed by cell surface biotinylation. However, no change in surface expression of KCNN4 following dexamethasone (15mins, 1 nM) or vehicle (15mins, methanol, 0.001% v/v) treatment was observed.

### 2.3 Expression of KCNQ1 channels in 16HBE14o^−^ airway epithelial cells

**Fig. 4:**
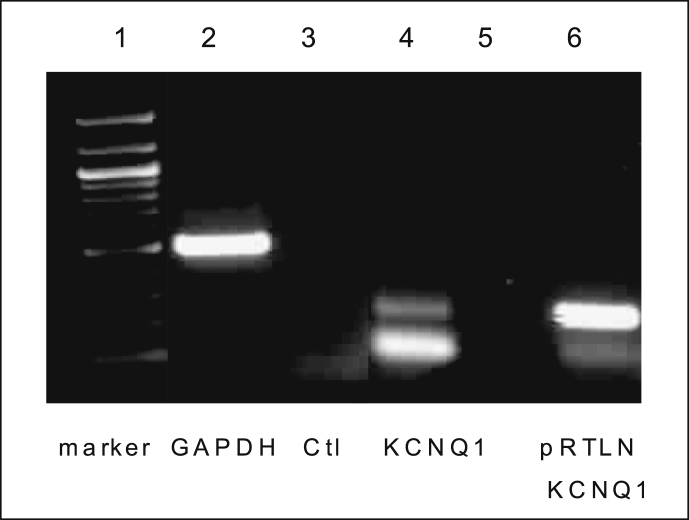
**Expression of KCNQ1 channel in 16HBE14o**^**−**^**cells.** Semi-quantitative RT-PCR analysis of KCNQ1 expression in 16HBE14o^−^ cells was amplified using specific primers for human KCNQ1, pRTLN-KCNQ1 (used as a positive control), ctl (negative control using hKCNQ1 primer pairs without cDNA), GAPDH (cDNA and GAPDH primer pairs) was used as a control.

**Fig. 5:**
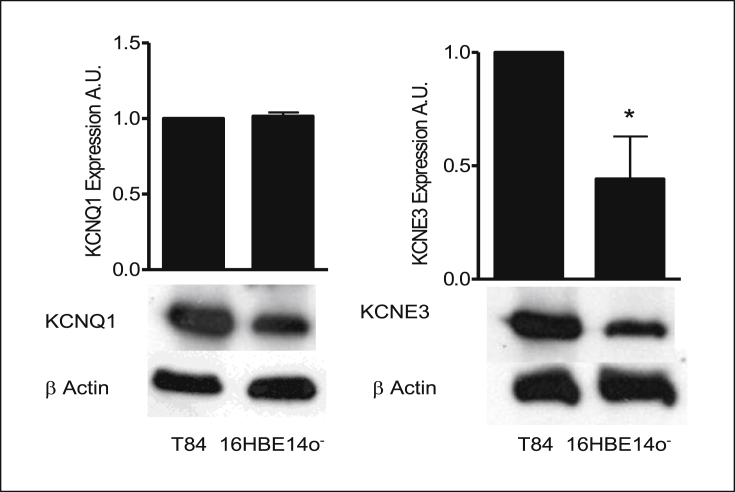
**KCNQ1 and KCNE3 are expressed in 16HBE14o**^**−**^**cells**. Western blot analysis of the KCNQ1 and KCNE3 proteins in 16HBE14o^−^ cells. Total protein (100 μg/lane) was transferred to nitrocellulose membrane after fractioning by SDS-PAGE and blotted with anti-KCNQ1 or anti-KCNE3. β-actin was used as a control to estimate protein loading.

Semi-quantitative RT-PCR was carried out to determine expression of KCNQ1. Different primer pairs, designed to specifically amplify human KCNQ1 was used to generate PCR products from cDNAs of 16HBE14o^−^ cells (Table 2). Human KCNQ1 (hKCNQ1) cloned into pTLN6, was kindly provided by Prof. Thomas Jentsch (Leibniz-Institute for Molecular Pharmacology, Berlin, Germany), and was used as a positive control for KCNQ1 expression. Agarose gels showing RT-PCR products amplified from 16HBE14o^−^ cDNA with PCR primer pairs for hKCNQ1. Fig. 4 shows that the hKCNQ1 product was detected in 16HBE14o^−^ cells. Western blot analysis was performed to determine expression of the KCNQ1 channel and its regulatory subunit KCNE3 in 16HBE14o^−^ cells (Fig. 5). As illustrated, anti-KCNQ1 and anti-KCNE3 antibodies recognized prominent bands with the molecular masses of 37 kDa and 27 kDa respectively. The T84 cell line was used as a positive control as these cells are known to express KCNQ1 protein. These data confirm that the cAMP-dependent KCNQ1 channels and their KCNE3 regulatory subunit are expressed in 16HBE14o^−^ cells.

**Table 2:** Oligonucleotide sequences of human KCNQ1 and GAPDH primers used for RT-PCR. HKCNQ1primers were designed using the Primer 3 program.

Protein	Primer Sequence 5′ → 3′	Product size bp
KCNQ1 (F)	ATCTGCGTAGCTGCCAAAC	272
KCNQ1 (R)	TAGCTCAAACCGTCGATGCG	
GAPDH (F)	CATTGGGGGTAGGAACACGGA	373
GAPDH(R)	GCCAAAAGGGTCATCATCTCCG

### 2.4 Expression of K_ATP_ channels in 16HBE14o^−^ epithelial cells

**Fig. 6:**
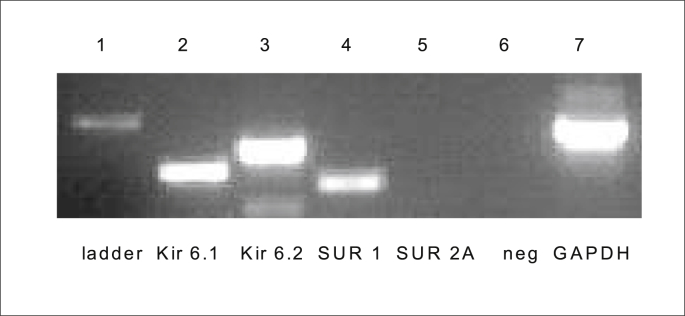
Agarose gel showing RT-PCR products amplified from 16HBE14o^−^ cDNA with PCR primer pairs for Kir6.1, Kir 6.2, SUR1, SUR2A and GAPDH. Semi-quantitative RT-PCR analysis of Kir6.1, Kir 6.2, SUR1, SUR2A and GAPDH expression in human bronchial epithelial cells was amplified using specific human primers.

The molecular identity of the lung K_ATP_ channel subunits was investigated in the 16HBE14o^−^ cells. Different primer pairs, designed to specifically amplify human Kir 6.1, Kir 6.2, SUR 1 and SUR 2A (Table 3) were used to generate PCR products from cDNAs of 16HBE14o^−^ cells. The Kir 6.1 and Kir 6.2 primer pairs amplified bp products, respectively (Fig. 6). In addition, the SUR 1 primer pairs amplified a 340 bp product. No product could be detected with primers for SUR2A. These results suggest that the K_ATP_ channels in 16HBE14o^−^ cells could be formed from Kir 6.1, Kir 6.2 and SUR2A subunits. The sulfonylurea receptors SUR 2A and subunits Kir 6.1 and Kir 6.2 were found to be expressed in the 16HBE14o^−^ cell line.

**Table 3:** Oligonucleotide sequences of primers used for K_ATP_ channel RT-PCR.

Protein	Primer Sequence 5′ → 3′	Product size bp
Kir 6.1(F)	GCCAGAAAGAGTATCATCCCGGAG	352
Kir 6.1(R)	CATTCCACTTTTCTCCATGTAAGC	
Kir 6.2 (F)	ATGCTGTCCCGCAAGGGCATC	360
Kir 6.2 (R)	TAGTCACTTGGACCTCAATGGAG	
SUR1 (F)	CGATGCCATCATCACAGAAG	340
SUR1(R)	CTGAGCAGCTTCTCTGGCTT	
SUR2A (F)	GCTGAAGAATATGGTCAAATCTC	355
SUR2A (R)	TGGAGTGTCATATTCTAAAATA	
GAPDH (R)	GCCAAAAGGGTCATCATCTCCG	373
GAPDH (F)	CATTGGGGGTAGGAACACGGA

### 2.5 Cell surface expression of KCNQ1

**Fig. 7:**
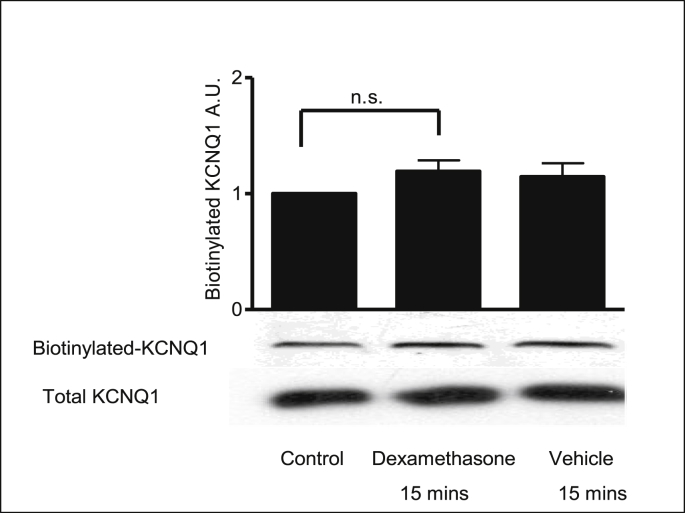
**The KCNQ1 channel expression at the basolateral membrane in 16HBE14o**^**−**^**cells.** Representative Western blot of labelling cell surface KCNN4 proteins with Biotin-LC-hydrazide. Dexamethasone (1nM) and vehicle control (methanol, 0.001% v/v) were added to 16HBE14o^−^ cells for 15 mins. Bar chart summary for the effect of dexamethasone on cell surface biotinylation in 16HBE14o^−^ cells. Values are mean ± SEM; n.s. denotes that values were not significant compared to control values. Statistical analysis was performed by one-way ANOVA followed by Tukey's multiple comparison tests.

Biotinylation experiments were performed to investigate the effect of dexamethasone on the localization of the KCNQ1 channel. The presence of KCNQ1 at the membrane was confirmed by cell surface biotinylation (Fig. 7). There was no change in surface expression of KCNQ1 following dexamethasone (1 nM) or vehicle (0.001% methanol) treatment indicating that dexamethasone does not change the cellular localization or expression of the KCNQ1 channel.

### 2.6 Effects of dexamethasone on expression and activation of PKC isoforms in human bronchial epithelial cells

**Fig. 8:**
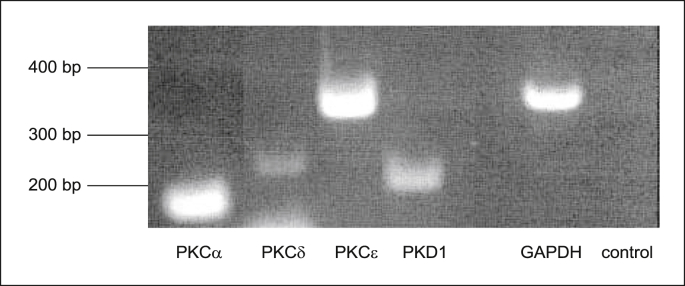
**Semi-quantitative RT-PCR analysis of PKC isoforms in human bronchial epithelial cells.** Total RNA was isolated from 16HBE14o^−^ cells using RNA easy kit (Qiagen). cDNA was reverse transcribed using poly dT primers and ImProm*™ Reverse Transcriptase System* (Promega, USA) and 1 μl of this reaction was directly amplified using GoTaq® Green Master Mix. (Promega, USA) using specific primers for human PKC isoforms and PKD (Table 3) and synthesised by MWG Biotech (Germany). The PCR reaction produced DNA fragments at the expected length for PKCα, PKCδ, PKCε and PKCμ (PKD1). GAPDH (+) (cDNA and GAPDH primer pairs) was used as a control. Image representative of three independent experiments.

Candidate protein kinase C isoforms of the cPKC and the nPKC groups were investigated in mediating the rapid anti-secretory responses to dexamethasone. Primers specific for PKCα, PKCδ, PKCε and PKD were designed using the Primer 3 program. PCR products of the expected size were generated from cDNA extracted from 16HBE14o^−^ cells (Table 4, Fig. 8).

**Table 4:** List of primers for PKC isoforms and GAPDH.

Protein	Primer Sequence 5′ → 3′	Product size bp
PKCα (F)	CGAGGAAGGAAACATGGAACTCAG	193
PKCα (R)	TTTCCACTACGAACGGCTCTCC	
PKCδ (F)	GCATCGCCTTCAACTCCTATGAGCT	249
PKCδ (R)	ACACACCCACGGTCACCTCAGA	
PKCε (F)	TCAATGGCCTTCTTAAGATCAAAA	388
PKCε (R)	CCTGAGAGATCGATGATCACATAC	
PKD (F)	TATCCAGGAAGGCGATCTTATTGAAGTG	236
PKD (R)	GCCTCACACCGCTGCAATTGTTG	
GAPDH (F)	CATTGGGGGTAGGAACACGGA	373
GAPDH (R)	GCCAAAAGGGTCATCATCTCCG

### 2.7 Expression of PKC isoforms in human bronchial epithelial cells

The results obtained from RT-PCR analysis were confirmed by western blotting. Western blots were performed on three independently derived cell lysates to establish PKC isoform expression. As a positive control lysates from MCF-7 breast cancer cell line was used. Western blot analysis revealed expression of these selected isoforms in 16HBE14o^−^ cells. An equivalent amount of protein (50 μg) was loaded in each track and equal loading of samples was confirmed by probing the same blot with β-actin monoclonal antibody.

**Fig. 9:**
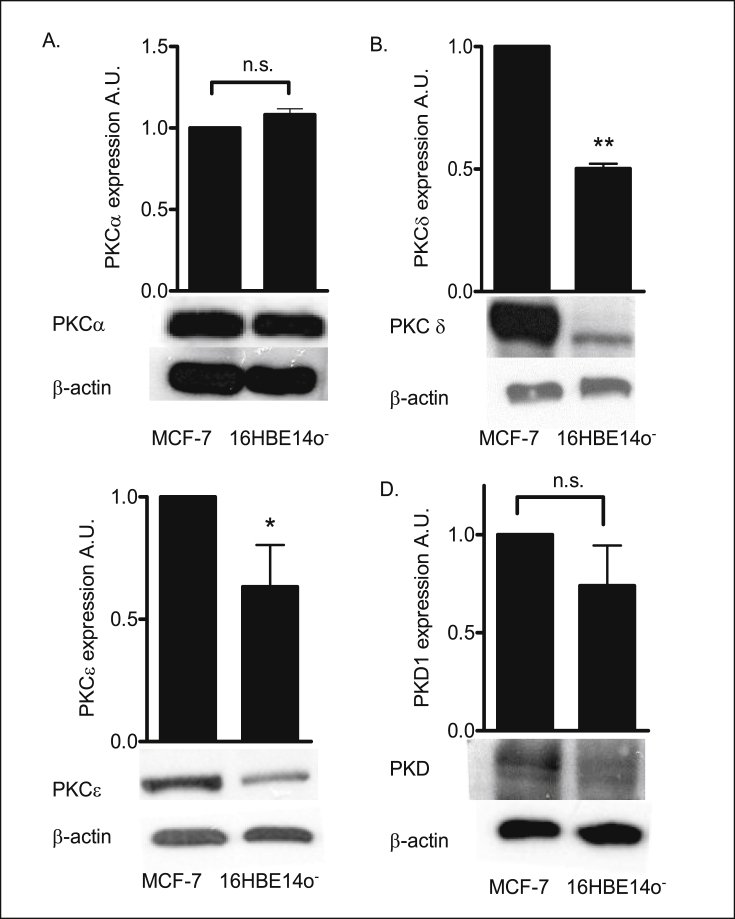
**PKCα, PKCδ, PKCε and PKD1 (PKCμ) are expressed in 16HBE14o**^**−**^**cells.** Representative Western blot analysis of PKC subunits: PKCα (Α), PKCδ (Β), PKCε (C) and PKD1 (D) in cellular extracts of 16HBE14o^−^ and MCF-7 cells. Total protein (50 μg/lane) was transferred to nitrocellulose membranes after fractionating by SDS-PAGE and blotted with anti-PKC antibodies. β-actin (42 kDa) was used as an internal control to estimate protein loading. The graphs represent densitometric analysis of PKC expression. Values are given as reflective PKC expression in 16HBE14o^−^ cell lysates compared to MCF- 7. Values are displayed as mean ± SEM (n = 3). ** Denotes p < 0.001, * denotes p < 0.01, n.s. denotes not significant (p > 0.05) between PKC isoform in MCF-7 and 16HBE14o^−^. Statistical analysis was performed using the Students paired *t*-test.

Immunoblots using antibodies for individual isoforms of PKC were performed: PKCα (Fig. 9Α), PKCδ (Fig. 9Β), PKCε (Fig. 9C) and PKD (Fig. 9D) in 16HBE14o^−^ cells and MCF-7 cells. Western blot analysis revealed the expression of the classical isoform PKCα (80 kDa), the novel isoforms PKCδ (78 kDa) and PKCε (95 kDa) and also expression of PKD (115 kDa). PKCα and PKD1 were expressed in equal quantities in 16HBE14o^−^ cells compared to MCF-7 cells (positive control). PKCδ and PKCε were significantly (**p < 0.001, *p < 0.01) respectively, less expressed in 16HBE14o^−^ cells compared to MCF-7 control. This reflected non-uniform expression of PKC isoform levels (PKCα > PKD1 > PKCε > PKCδ levels of expression).

### 2.8 Effect of dexamethasone on PKCα, PKCδ and PKCε activity in 16HBE14o^−^ cells

The levels of PKC isoform activation can be observed through changes in phosphorylation states at key amino acid residues. For PKCα activation Ser657 autophosphorylation in the hydrophobic *C*-terminal is required for catalytic activation and stabilisation of the protein upon translocation to the plasma membrane. PKCδ activity was assessed using an antibody to PKCδ phosphorylated at Ser643. PKCδ autophosphorylates at Ser643 in the turn motif after initial phosphorylation at Thr505 by PDK-1 in the activation loop. PKCε activation requires phosphorylation at three sites (1): Thr666 in the activation loop (2), Ser729 in the *C*-terminal hydrophobic region and (3) autophosphorylation at Thr710. The level of phosphorylation at these residues can be used as a measure of catalytic activity of the kinase isoforms. By employing antibodies specific to the phosphorylated form of PKCs, activation was monitored in response to dexamethasone by Western blot analysis.

**Fig. 10:**
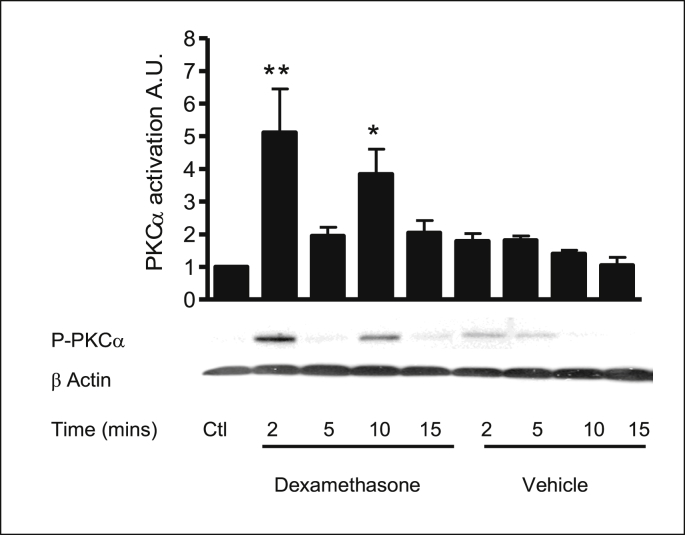
**PKCα is rapidly activated in response to dexamethasone in 16HBE14o**^**−**^**cells.** Representative Western blot analysis of phospho-PKCα in cellular extracts of 16HBE14o^−^. The activation of PKCα by dexamethasone was monitored using antibodies specific to the phosphorylated form of PKCα (Ser 657). β-actin (42 kDa) was used as an internal control to estimate protein loading. The graph represents densitometric analysis at specific time points of dexamethasone treatment. Values are given as fold changes in PKCα activation of 16HBE14o^−^ cell lysates. Values are displayed as mean ± SEM (n = 4). ** Denotes significance: p < 0.001, * denotes p < 0.01 between control and treated values. Statistical analysis was performed using one-way ANOVA followed by Tukey's multiple comparison tests.

**Fig. 11:**
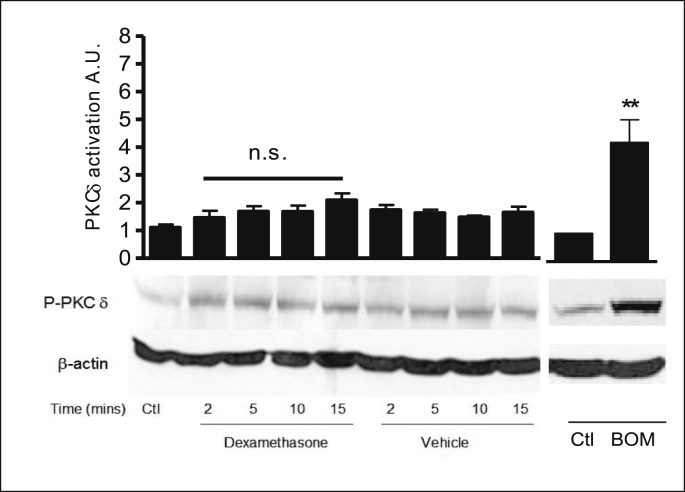
**Dexamethasone does not activate the nPKC isoform: PKCδ in 16HBE14o**^**−**^**cells.** Representative Western blot analysis of phospho-PKCδ in cellular extracts of 16HBE14o^−^. Using antibodies specific to the phosphorylated form of PKCδ (Ser 643), the activation by dexamethasone was monitored by Western blot analysis. β-actin (42 kDa) was used as an internal control to estimate protein loading. The graph represents densitometric analysis at specific time points of dexamethasone treatment. Bombesin (BOM) was used as a positive control for PKCδ activity. Values are given as fold changes in PKCδ activation of 16HBE14o^−^ cell lysates. Values are displayed as mean ± SEM (n = 4); n.s. denotes no significance between control and treated values. Statistical analysis was performed using one-way ANOVA followed by Tukey's multiple comparison tests.

**Fig. 12:**
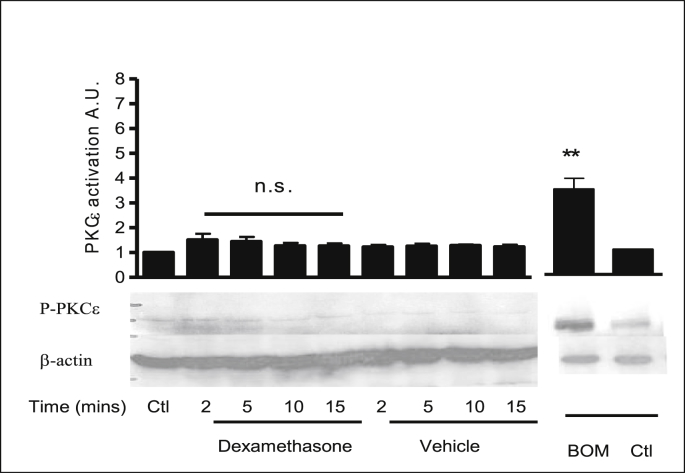
**Dexamethasone does not activate the nPKC isoform: PKCε in 16HBE14o**^**−**^**cells.** Representative Western blot analysis of phospho- PKCε in cellular extracts of 16HBE14o^−^. Using antibodies specific to the phosphorylated form of PKCε (Ser 729), the activation by dexamethasone was monitored by Western blot analysis. β-actin (42 kDa) was used as an internal control to estimate protein loading. The graph represents densitometric analysis at specific time points of dexamethasone treatment. Values are given as fold changes in PKCε activation of 16HBE14o^−^ cell lysates as detailed in Table 7. Bombesin (BOM) was used as a positive control for PKCε activity. Values are displayed as mean ± SEM (n = 3); n.s. denotes no significance between control and treated values. Statistical analysis was performed using one-way ANOVA followed by Tukey's multiple comparison tests.

PKCα activation was assessed by probing with a specific antibody to phosphorylation at Ser 657. Dexamethasone treatment produced a biphasic activation of PKCα at 2 and 10 mins (Fig. 10, Table 5). PKCδ activity was measured using a specific antibody to phosphorylation at Ser643. Dexamethasone treatment had no effect on PKCδ phosphorylation levels (Fig. 11, Table 6). PKCε activity was measured using a specific antibody to phosphorylation at Ser 729. Dexamethasone treatment had no effect on PKCε phosphorylation levels (Fig. 12, Table 6). Bombesin was used as a positive control for PKC activation. Stripping the blot and reprobing with β-actin showed equal loading of all lanes. These results show that dexamethasone selectively activates classical PKCα and does not rapidly activate the novel PKC isoforms: PKCδ and PKCε.

**Table 5:** Summary of the fold increase in PKCα activity induced by dexamethasone.

Treatment	PKCα activation (phosphorylation at Ser 657)
Lysate control	1 ± 0
Dexamethasone (1nM, 2 min)	5.12 ± 1.9
Dexamethasone (1nM, 5 min)	1.95 ± 0.39
Dexamethasone (1nM, 10 min)	3.84 ± 0.39
Dexamethasone (1nM, 15 min)	2.05 ± 0.56
Methanol (0.001% v/v, 2 min)	1.79 ± 0.33
Methanol (0.001% v/v, 5 min)	1.81 ± 0.20
Methanol (0.001% v/v, 10 min)	1.40 ± 0.16
Methanol (0.001% v/v, 15 min)	1.05 ± 0.34

**Table 6:** Summary of the fold increases in PKCδ activity.

Treatment	PKCδ activation (phosphorylation at Ser 643)
Lysate control	1.1 ± 0.27
Dexamethasone (1nM, 2 min)	1.46 ± 0.36
Dexamethasone (1nM, 5 min)	1.69 ± 0.27
Dexamethasone (1nM, 10 min)	1.67 ± 0.32
Dexamethasone (1nM, 15 min)	2.09 ± 0.32
Methanol (0.001% v/v, 2 min)	1.74 ± 0.26
Methanol (0.001% v/v, 5 min)	1.89 ± 0.40
Methanol (0.001% v/v, 10 min)	1.78 ± 0.46
Methanol (0.001% v/v, 15 min)	1.65 ± 0.31
Control	1.43 ± 0.28
Bombesin	4.03 ± 0.12

**Table 7:** Summary of the fold increase in PKCε activity induced by dexamethasone.

Treatment	PKCε activation (phosphorylation at Ser 729)
Lysate control	1 ± 0
Dexamethasone (1nM, 2 min)	1.5 ± 0.31
Dexamethasone (1nM, min)	1.45 ± 0.24
Dexamethasone (1nM, 10 min)	1.27 ± 0.15
Dexamethasone (1nM, 15 min)	1.26 ± 0.13
Methanol (0.001% v/v, 2 min)	1.22 ± 0.11
Methanol (0.001% v/v, 5 min)	1.25 ± 0.12
Methanol (0.001% v/v, 10 min)	1.29 ± 0.05
Methanol (0.001% v/v, 15 min)	1.22 ± 0.11
Bombesin	3.54 ± 0.24
Control	1.71 ± 0.17

### 2.9 Expression of cAMP-adenylyl cyclase -PKA signaling pathway in 16HBE14o^−^ cells

**Fig. 13:**
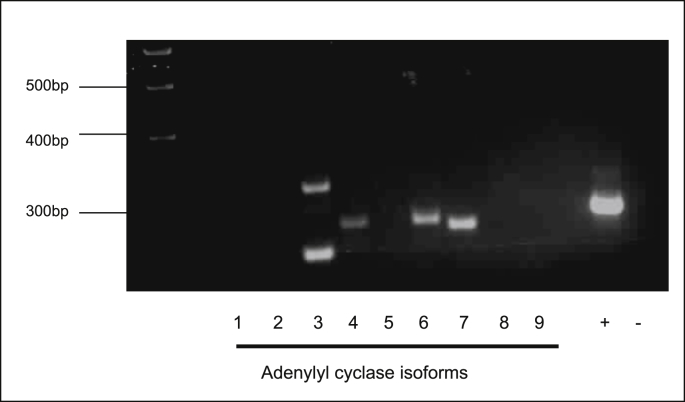
**Semi-quantitative RT-PCR analysis of adenylyl cyclase isoforms AC1- 9 in human bronchial epithelial cells**. Total RNA was isolated from 16HBE14o^−^ cells using RNA easy kit (Qiagen). cDNA was reverse transcribed using poly dT primers and ImProm*™ Reverse Transcriptase System* (Promega, USA) and 1 μl of this reaction was directly amplified using GoTaq® Green Master Mix. (Promega, USA) using specific primers for human AC isoforms (Xu, D, Isaaca, C (2001)) (Table 3) and synthesised by MWG Biotech (Germany). The PCR reaction produced DNA fragments at the expected length for AC 3, 4, 6 and 7. (+) denotes GAPDH and (−) denotes negative control. Figure representative of three independent experiments.

Adenylyl cyclase (AC) expression was determined in 16HBE14o^−^ cells. Because of the unavailability of satisfactory antibodies for most of the AC isoforms, the expression of AC isoforms was investigated by RT- PCR (Table 8). As shown in Fig. 13, the AC isoforms AC3, AC4, AC6 and AC7 were found to be expressed in 16HBE14o^−^ cells.

**Table 8:** List of primers for human adenylyl cyclase isoforms AC1-9 and GAPDH.

Protein	Primer Sequence 5′ → 3′	Product size bp	GenBank Accession No.
AC1 (F)	CATGACCTGCGAGGACGAT	446	L05500
AC1 (R)	ACAGGAGACTGCGAATCTGAA		
AC2 (F)	GGGGCTGCGTTTCTCT	369	X74210
AC2 (R)	CAGGAACACGGAACAGGATA		
AC3 (F)	CACGGGACCCAGCAAT	263	NM_004036
AC3 (R)	GCTCTAAGGCCACCATAGGTA		
AC4 (F)	TGAACCATGGACCCGTAG	287	AF088070
AC4 (R)	GCGAGTGCAATCTCAGC		
AC5 (F)	ACCAAGGCTACACTCAACTAC	163	U65473
AC5 (R)	GGTTCATCTTGGCGATCA		
AC6 (F)	GGCATTGATGATTCCAGCAAAGAC	380	AB007882
AC6 (R)	TGCAGGGCCTTAGGGAACAGA		
AC7 (F)	TTAGCACATGATGAAAACAGACTT	359	NM_001114
AC7 (R)	CACTGGAGGGAAGAGATTTATG		
AC8 (F)	CGGGATTTGGAACGCCTCTA	543	NM_001115
AC8 (R)	CCGGTCTGACAGGTAACTGATAA		
AC9 (F)	CACCGCAAAATACTTAGATGACG	497	NM_001116
AC9 (R)	CCTTCTCCTGCAAGATCTCACAC		
GAPDH (F)	CATTGGGGGTAGGAACACGGA	373	XO2231
GAPDH (R)	GCCAAAAGGGTCATCATCTCCG

### 2.10 Expression of PKA regulatory and catalytic subunits in human bronchial epithelial cells

**Fig. 14:**
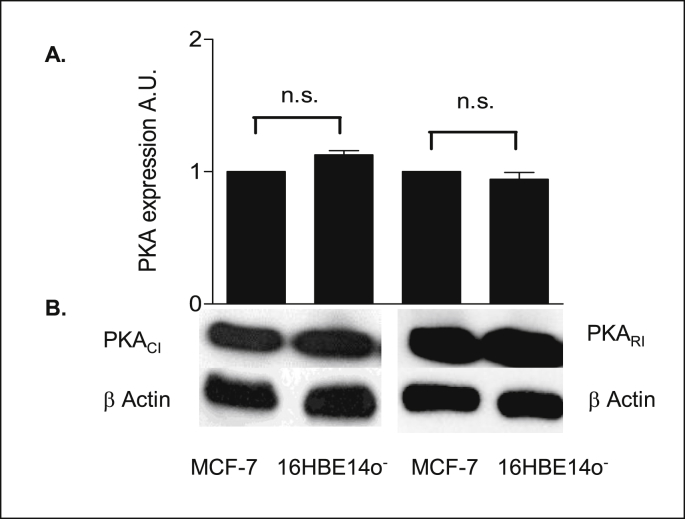
**PKA**_**CI**_**and PKA**_**RI**_**are expressed in 16HBE14o**^**−**^**cells. A:** The graph represents densitometric analysis of PKA expression. Values are given as fold differences in PKA expression between MCF-7 and 16HBE14o^−^ cell lysates. Values are displayed as mean ± SEM (n = 3). n.s. denotes not significant (P > 0.05) between MCF-7 and 16HBE14o^−^ cells in PKA_CI_ and PKA_RI_ expression. Statistical analysis was performed by the Student's paired *t*-test for three independent experiments. **B:** Representative Western blot analysis of PKA_CI_ and PKA_RI_ subunit in cellular extracts of 16HBE14o^−^ and MCF-7 cells. Total protein (50 μg/lane) was transferred to nitrocellulose membranes after fractionating by SDS-PAGE and blotted with anti-PKA_RI_ (48kDa) and anti-PKA_CI_ (40kDa). β-actin (42 kDa) was used as an internal control to estimate protein loading.

Since AC isoforms are expressed in 16HBE14o^−^ cells, it was of interest to investigate the expression levels of the catalytic and regulatory subunits of PKA in 16HBE14o^−^ cells. The PKA isoform I (PKA_I_) the soluble cytosolic isoform, was investigated in cellular extracts in this study, as distinct from isoform II which is membrane bound. Total untreated cellular lysates of 16HBE14o^−^ cells were prepared and subjected to western blot analysis by probing with specific antibodies to endogenous levels of PKA regulatory (PKA_RI_) and catalytic (PKA_CI_) subunits. As shown in Fig. 14, 16HBE14o^−^ cells express equal levels of both PKA_RI_ and PKA_CI_ subunits compared with MCF-7 cells used as a positive control. Expression differences were normalized for loading by probing for total β-actin levels.

### 2.11 Dexamethasone effects on cAMP-dependent protein kinase A activity

**Fig. 15:**
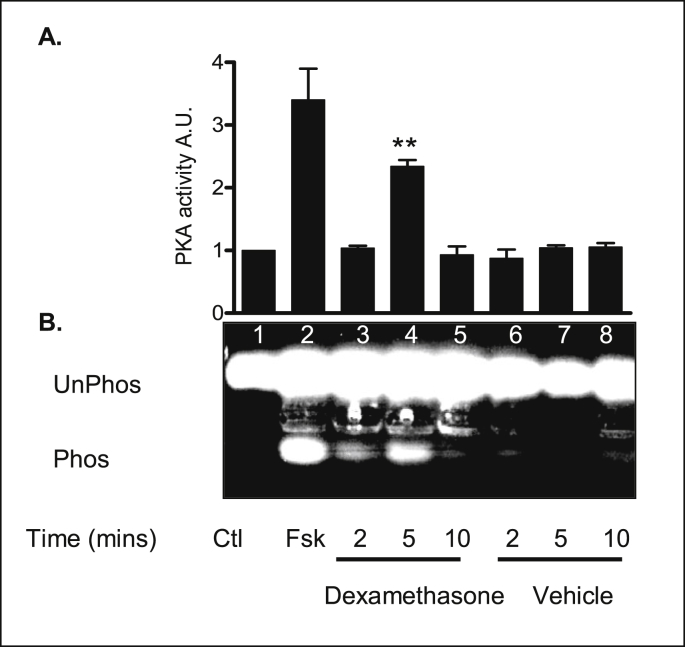
**PKA is rapidly activated in response to dexamethasone in 16HBE14o**^**−**^**cells. A:** The graph represents densitometric analysis at specific time points of dexamethasone treatment. Values are given as fold changes in PKA phosphorylation of the F-Kemptide PepTag for 16HBE14o^−^ cell lysates. Values are displayed as mean ± SEM (n = 6). ** Denotes significance (p < 0.001) between control and treated values. Statistical analysis was performed by one-way ANOVA followed by Tukey's multiple comparison tests. **B:** Representative image of PKA activation of an F-Kemptide PepTag in cellular extracts from 16HBE14o^−^ cells. PKA activity phosphorylated the F-Kemptide PepTag peptide changing its net charge from +1 to −1. This allows the phosphorylated and nonphosphorylated forms of the substrate to be rapidly separated on agarose gel. Lane 1, lysate control, Lane 2, forskolin (20 μM/5 min), Lane 3, dexamethasone (1 nM, 2 min), Lane 4, dexamethasone, (1 nM, 5 min), Lane 5, dexamethasone (1 nM, 10 min), Lane 6, vehicle control (methanol 0.001% v/v/2 min), Lane 7, vehicle control (methanol 0.001% v/v, 5 min), Lane 8, vehicle control (methanol 0.001% v/v, 10 min).

The cAMP-adenylyl-PKA signaling pathway is clearly expressed in 16HBE14o^−^ cells. We therefore examined the effects of dexamethasone on PKA activity. To determine the time course of dexamethasone activation of PKA in 16HBE14o^−^ cells, serum starved cells were exposed to dexamethasone (1 nM) or equivalent vehicle (methanol, 0.001% v/v) for the duration of 2, 5 and 10 mins. PKA activity was rapidly and dramatically upregulated after 5 mins in response to dexamethasone (1 nM) treatment and returned to basal levels after 10 mins (Fig. 15). The effect was comparable to forskolin (20 μM) a well-known activator of AC. A summary of the fold increases in PKA activity for each treatment is shown in Table 9.

**Table 9:** Summary of the fold increases in PKA activity following dexamethasone treatment.

Treatment	Fold increase in PKA activity
Forskolin (20 μM, 5 min)	3.4 ± 0.81
Dexamethasone (1 nM, 0 min)	1 ± 0
Dexamethasone (1 nM, 2 min)	1.03 ± 0.05
Dexamethasone (1 nM, 5 min)	2.6 ± 0.35
Dexamethasone (1 nM, 10 min)	0.93 ± 0.17
Methanol (0.001% v/v, 2 min)	0.87 ± 0.25
Methanol (0.001% v/v, 5 min)	1.04 ± 0.05
Methanol (0.001% v/v, 10 min)	1.05 ± 0.08

### 2.12 PKA and cAMP effects on cAMP-dependent protein kinase activity

**Fig. 16:**
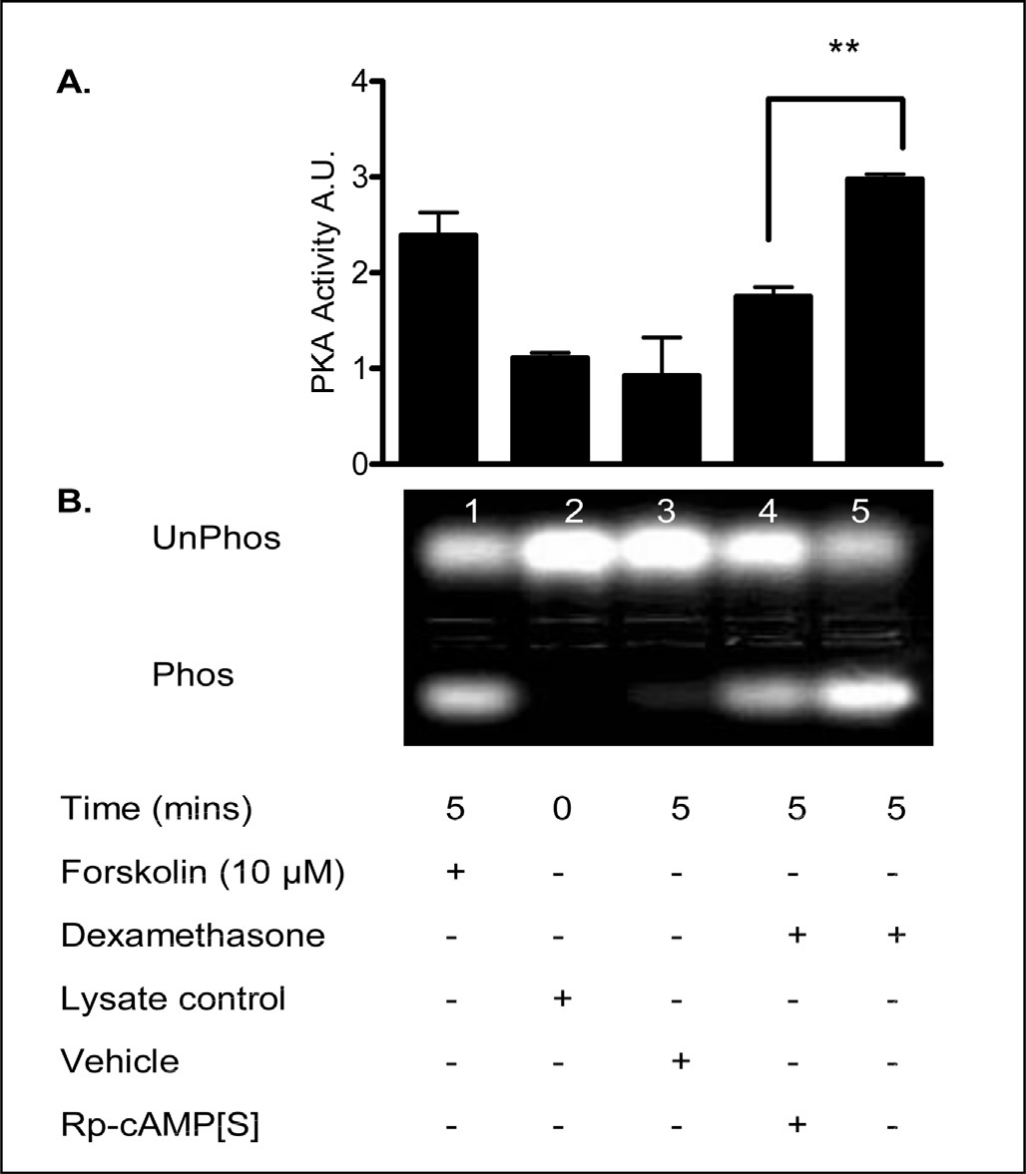
**Dexamethasone effect on PKA activity in 16HBE14o**^**−**^**cells**. **A:** The graph represents densitometric analysis of treatments. Values are given as fold changes in PKA activity, measured as phosphorylation of the F-Kemptide PepTag for 16HBE14o^−^ cell lysates. Values are displayed as mean ± SEM (n = 4). ** Denotes significance (p < 0.001) between dexamethasone and dexamethasone + Rp-cAMP[S] treated values. Statistical analysis was performed by one-way ANOVA followed by Tukey's multiple comparison tests. **B:** UV-illuminated agarose gel of the products of reactions run with F-Kemptide and 16HBE14o^−^ homogenate. PKA activity phosphorylated the F-Kemptide PepTag peptide changing its net charge from +1 to −1. This allows the phosphorylated and nonphosphorylated forms of the substrate to be rapidly separated on agarose gel. Lane 1, forskolin (20 μM/5 min), Lane 2, lysate control, Lane 3, vehicle control (methanol, 0.001% v/v, 5 min), Lane 4, Rp-cAMP [S] (prior treatment 20 μM/40 min) and dexamethasone (1 nM, 5 min), Lane 5, dexamethasone (1 nM, 5 min) (Verriere et al., 2005).

The adenylyl cyclase activator, forskolin, and the PKA antagonist, R_p_-cAMP[S], were used as positive and negative controls for PKA stimulation, respectively. Dexamethasone increased PKA activity and this effect was inhibited by R_p_-cAMP[S] (Fig. 16). The effect was comparable to forskolin, a known activator of adenylyl cyclase. Table 10 shows a summary of fold increases in PKA activity.

**Table 10:** Summary of the fold increases in PKA activity.

Treatment	Fold increase in PKA activity
Forskolin (20 μM, 5 min)	2.39 ± 0.30
Dexamethasone (1 nM, 5 min)	2.97 ± 0.07
Dexamethasone (1 nM, 5 min) + Rp-cAMP[S] (20 μM, prior treatment 40 min)	1.75 ± 0.13
Lysate control	1.1 ± 0.07
Methanol (0.001% v/v, 5 min)	0.92 ± 0.51

### 2.13 Dexamethasone induces rapid non-genomic PKA activation

**Fig. 17:**
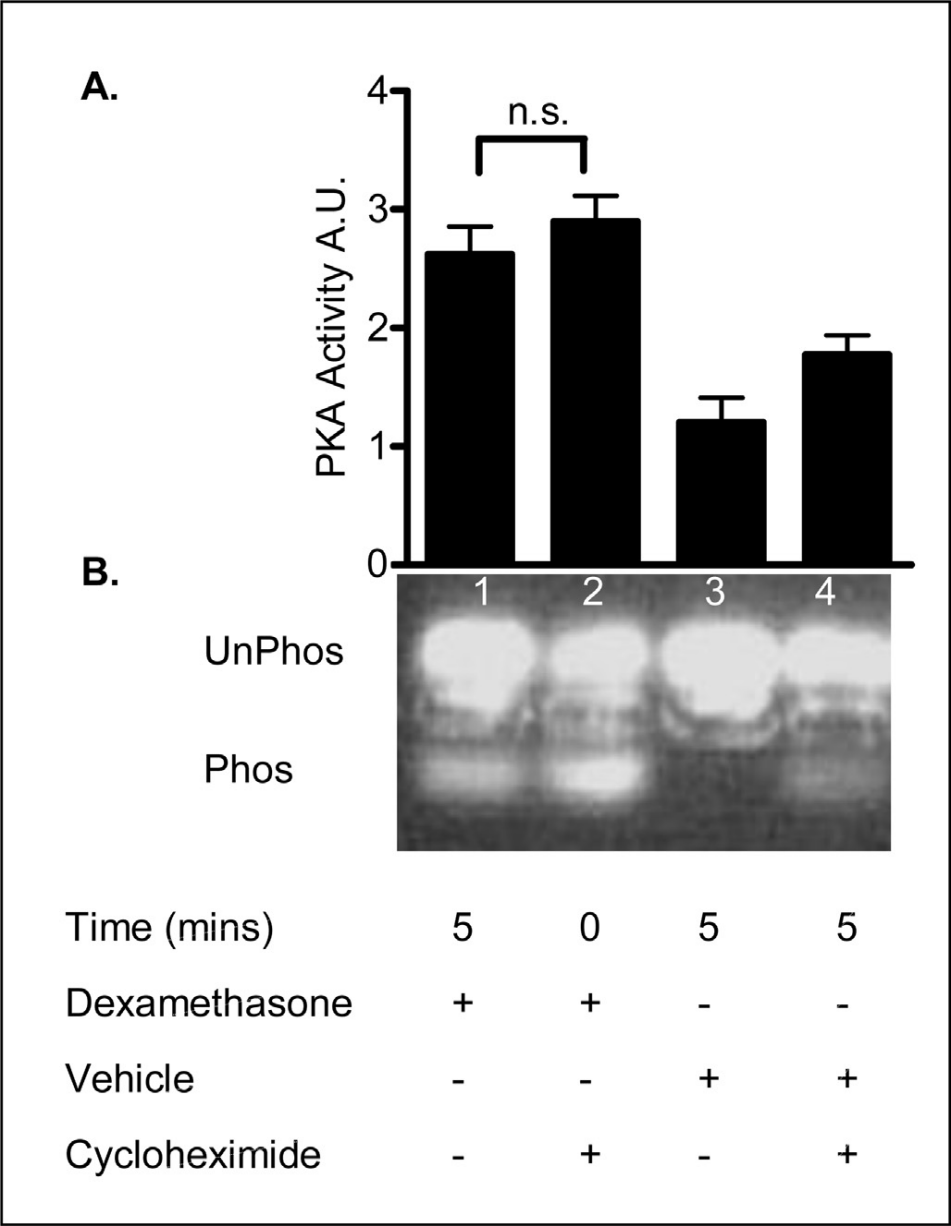
**Dexamethasone induces PKA activity independent of translation. A:** The graph represents densitometric analysis of treatments. Values are given as fold changes in PKA activity measured as phosphorylation of the F-Kemptide PepTag for 16HBE14o^−^ cell lysates. Values are displayed as mean ± SEM (n = 4), n.s. denotes not significance (p > 0.05) between dexamethasone and dexamethasone and inhibitor treated values. Statistical analysis was performed by one-way ANOVA followed by Tukey's multiple comparison tests. **B:** UV-illuminated agarose gel of the products of reactions run with F-Kemptide and 16HBE14o^−^ homogenate. Lane 1, dexamethasone (1 nM, 5 min) Lane 2, cycloheximide (prior treatment 1 μM/60 min) and dexamethasone, (1 nM, 5 min) Lane 3, vehicle control (methanol, 0.001% v/v, 5 min), Lane 4, methanol, 0.001% v/v 5 min and cycloheximide.

The rapid time course (5 minutes) of the dexamethasone effect on PKA activity suggests that this response does not involve a classical genomic mechanism. In order to verify this hypothesis we investigated the dexamethasone response in the presence of cycloheximide, an inhibitor of mRNA translation. Dexamethasone (1 nM) stimulated a 2.62 ± 0.29 -fold increase in total PKA activity in 16HBE14o^−^ cells after 5min (Fig. 17). This increase was not inhibited by preincubation with cycloheximide (1 μM) for 1 hour. The vehicle control and cycloheximide alone did not stimulate a significant change in PKA activity relative to untreated control. Table 11 shows a summary for the fold changes in PKA activity. This result indicates that the dexamethasone induced increase in PKA activity observed after 5 min treatment is not dependent on changes in gene translation.

**Table 11:** Summary of the fold increases in PKA activity.

Treatment	Fold increase in PKA activity
Dexamethasone (1nM, 5 min)	2.62 ± 0.29
Dexamethasone (1 nM, 5 min) + cycloheximide (1 μM, 60 min prior treatment)	2.9 ± 0.27
Methanol (0.001% v/v, 5 min)	1.2 ± 0.26
Methanol (0.001% v/v, 5 min) + cycloheximide (1 μM, 60 min prior treatment)	1.77 ± 0.21

### 2.14 Role of MR and GR in dexamethasone PKA response? - Effect of the classic glucocorticoid antagonist, RU486 and the mineralocorticoid antagonist, spironolactone on dexamethasone-induced PKA activity

**Fig. 18:**
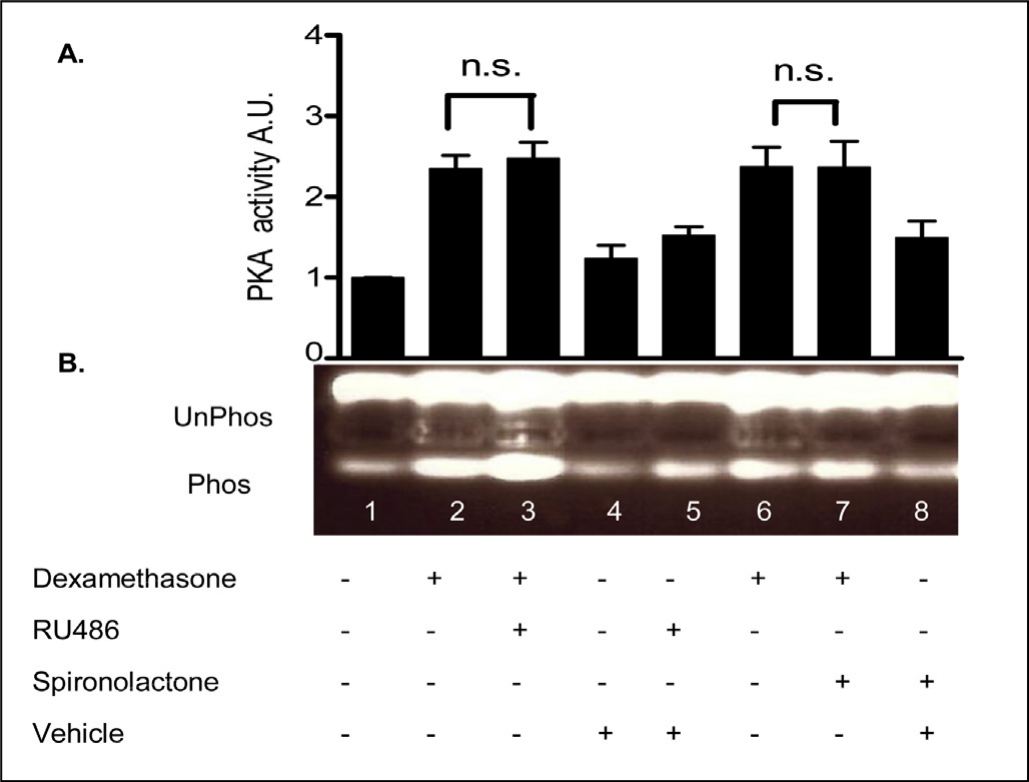
**Dexamethasone induces PKA activity independent of the classical GR and MR receptor antagonists. A:** The graph represents densitometric analysis at specific time points of dexamethasone treatment. Values are given as fold changes in PKA activity measured as phosphorylation of the F-Kemptide PepTag for 16HBE14o^−^ cell lysates. Values are displayed as mean ± SEM (n = 4). n.s. denotes not significant between dexamethasone and dexamethasone and inhibitor treated values. Statistical analysis was performed by one-way ANOVA followed by Tukey's multiple comparison tests. **B:** UV-illuminated agarose gel of the products of reactions run with F-Kemptide and 16HBE14o^−^ homogenate. Lane 1, lysate control, Lane 2, dexamethasone (1 nM, 5 min), Lane 3, RU486 (prior treatment 1 μM/30 min) and dexamethasone (1 nM, 5 min), Lane 4, vehicle control (methanol, 0.001% v/v, 5 min), Lane 5, RU486 + vehicle control (methanol 0.001% v/v, 5 min), Lane 6, dexamethasone (1 nM, 5 min), Lane 7, spironolactone (prior treatment 10 μM/30 min) and dexamethasone, (1 nM, 5 min), Lane 8, spironolactone + vehicle control (methanol 0.001% v/v, 5 min).

In order to investigate the receptor involved in the rapid response to dexamethasone, RU486 and spironolactone were used as antagonists of the GR and the MR, respectively. In control experiments the receptor antagonists did not affect basal PKA activity. Furthermore, neither RU486 nor spironolactone treatment significantly affected the dexamethasone induced PKA activation in 16HBE14o^−^ cells (Fig. 18, Table 12). Taken together, these data indicate the rapid effect of dexamethasone on PKA activity in human airway epithelial cells is a non-genomic response that does not involve the nuclear GR or MR receptors.

**Table 12:** Summary of the fold increases in PKA activity.

Treatment	Fold increase in PKA activity
Lysate control	1.0 ± 0
Dexamethasone (1nM, 5 min)	2.35 ± 0.17
Dexamethasone (1 nM, 5 min) + RU486 (1 μM, 30 min prior treatment)	2.48 ± 0.20
Methanol (0.001% v/v, 5 min)	1.24 ± 0.16
Methanol (0.001% v/v, 5 min) + RU486 (1 μM)	1.52 ± 0.11
Dexamethasone (1nM, 5 min)	2.37 ± 0.25
Dexamethasone + spironolactone (10 μM, 30 min prior treatment)	2.37 ± 0.33
Methanol (0.001% v/v, 5 min) + spironolactone (10 μM)	1.49 ± 0.21

### 2.15 Role of pertussis toxin sensitive G protein-coupled receptors in the PKA response to dexamethasone

**Fig. 19:**
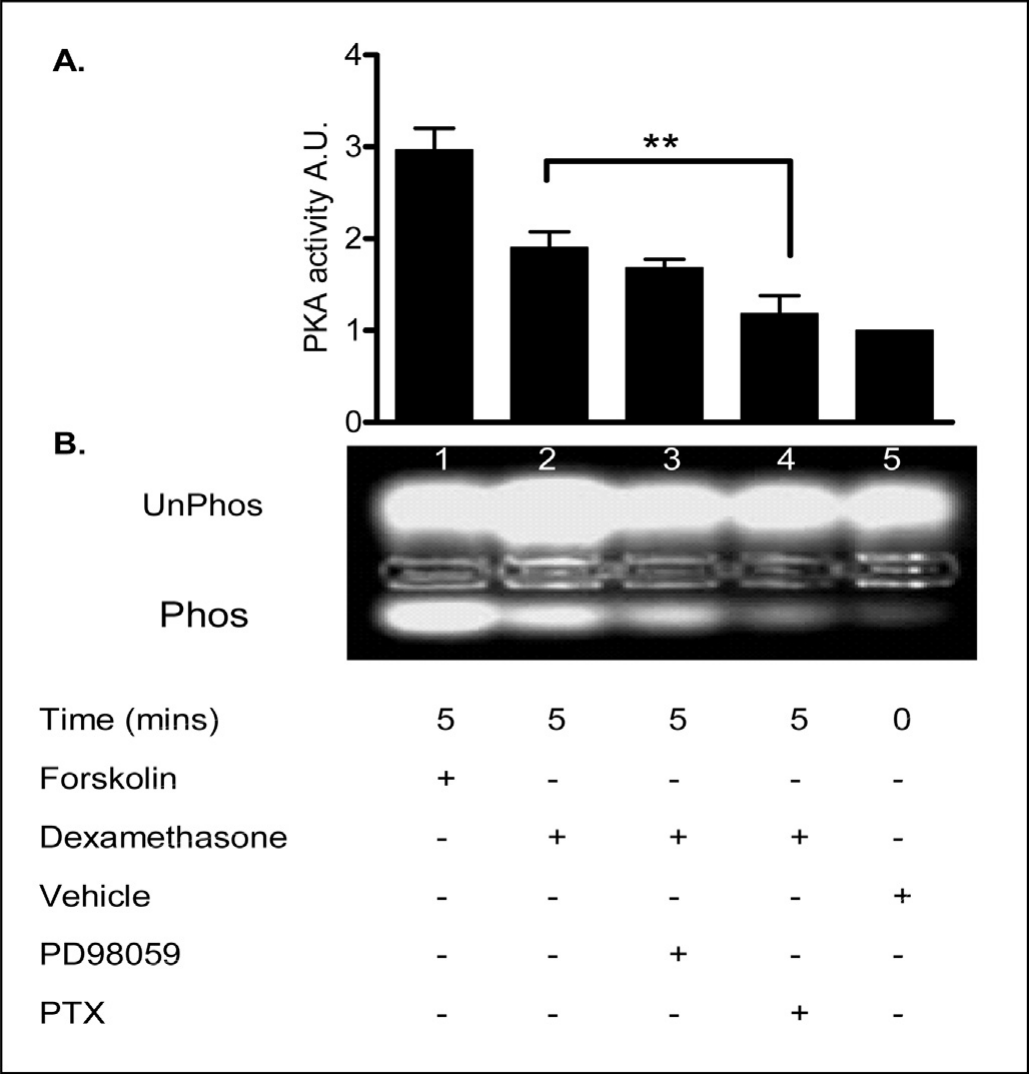
**Role of pertussis toxin sensitive G Protein–coupled receptor in the dexamethasone-induced PKA activity. A:** The graph represents densitometric analysis at specific time points of dexamethasone treatment. Values are given as fold changes in PKA activity measured as phosphorylation of the F-Kemptide PepTag for 16HBE14o^−^ cell lysates. Values are displayed as mean ± SEM (n = 4). ** Denotes significance (p < 0.001) between dexamethasone and dexamethasone + pertussis toxin treated values. Statistical analysis was performed by one-way ANOVA followed by Tukey's multiple comparison tests. **W** UV-illuminated agarose gel of the products of reactions run with F-Kemptide and 16HBE14o^−^ cell homogenate. PKA activity phosphorylated the PepTag peptide (F-Kemptide) changing its net charge from +1 to −1. *Lane 1*, forskolin (20 μM/5 min); *lane 2*, dexamethasone (1 nM/5 min); *lane 3*, PD98059 (prior treatment 50 μM/40 min) and dexamethasone (1 nM/5 min); *lane 4*, pertussis toxin (prior treatment 2 μg/l, 20 min) and dexamethasone (1 nM/5 min); *lane 5*, vehicle control (methanol 0.001% v/v), (Verriere et al., 2005).

The role of G protein-coupled receptors (GPCRs) in the rapid response to dexamethasone was investigated using pertussis toxin (PTX). As shown in Fig. 19 and Table 13, PKA activity was up-regulated in less than 5 min by 40% over control (p < 0.01) following 1 nM dexamethasone treatment. The G_i_ protein inhibitor, PTX (2 μg/l) significantly inhibited the activation of PKA by dexamethasone. The cells pretreated with PTX before dexamethasone did not show a significantly increased phosphorylation compared with untreated control cells (*p* > 0.1). In contrast, the MEK 1 inhibitor, PD98059 (50 μM), had no significant effect on PKA activation by the steroid (p > 0.1). In these experiments, the AC activator, forskolin, and the PKA antagonist, (*R*_p_)-cAMP, were used as positive and negative controls for PKA stimulation, respectively. The vehicle control (methanol 0.001% v/v) did not stimulate PKA activity. These data suggest a role for a PTX sensitive G protein-coupled receptor in the rapid PKA response to dexamethasone.

**Table 13:** Summary of the fold increases in PKA activity.

Treatment	Fold increase in PKA activity
Forskolin (20 μM, 5 min)	2.97 ± 0.24
Dexamethasone (1nM, 5 min)	1.9 ± 0.17
Dexamethasone (1 nM, 5 min) + PD98059 (50 μM, 30 min prior treatment)	1.68 ± 0.09
Dexamethasone (1 nM, 5 min) + PTX (2 μg/l, 20 min prior treatment)	1.18 ± 0.20
Methanol (0.001% v/v, 5 min)	1 ± 0

### 2.16 Activation of the ERK1/2 MAPK pathway by dexamethasone

**Fig. 20:**
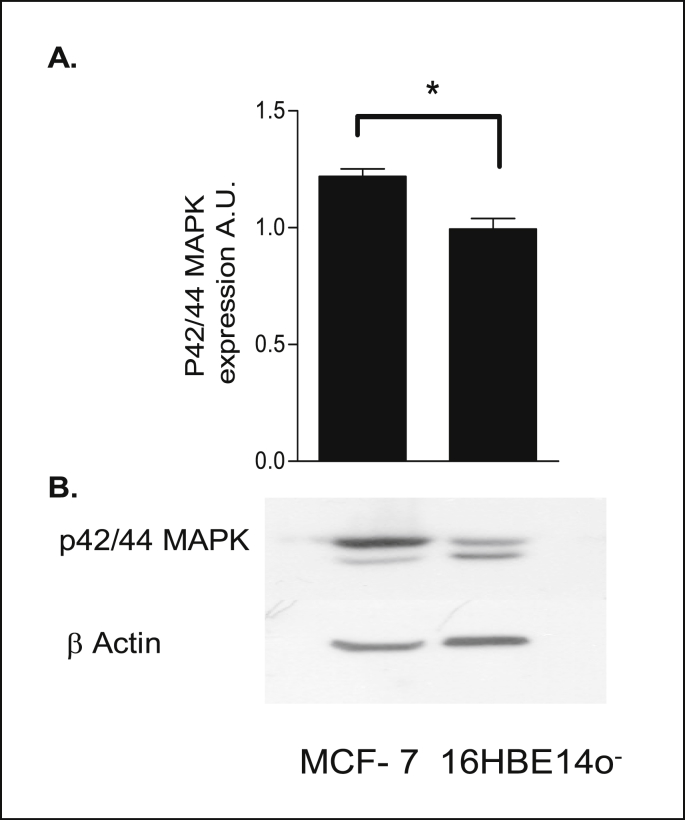
**ERK1/2 MAPK is expressed in 16HBE14o**^**−**^**cells. A:** The graph represents densitometric analysis of MAPK expression. Values are given as fold changes in ERK1/2 MAPK expression in MCF- 7 and 16HBE14o-cell lysates. Values are displayed as mean ± SEM (n = 3). Statistical analysis: Student's paired *t*-test. **B:** Representative Western blot analysis of ERK1/2 MAPK in cellular extracts of 16HBE14o- and MCF-7 cells. Total protein (30 μg/lane) was transferred to nitrocellulose membranes after fractionating by SDS-PAGE and blotted with anti-ERK1/2 MAPK. β-actin (42 kDa) was used as an internal control to estimate protein loading.

PKA is known to activate downstream ERK1/2 MAPK signaling to induce non-genomic responses to steroid hormones. The basal expression level of ERK1/2 MAPK in 16HBE14o^−^ cells was examined. Total untreated cellular lysates of 16HBE14o^−^ cells were prepared, subjected to western blot analysis and probed using a specific antibody recognising ERK1/2 MAPK. Expression differences were normalized for loading by probing for total β-actin levels. MCF-7 cells were used as a positive control as they are known to express ERK1/2 MAPK. This result clearly showed that ERK1/2 MAPK was expressed in 16HBE14o^−^ cells (Fig. 20).

### 2.17 Dexamethasone activation of ERK1/2 MAPK

The effect of dexamethasone on the activation of ERK1/2 MAPK was next examined. Phosphorylation of ERK1/2 MAPK on key threonine 202 and tyrosine 204 residues is strongly correlated with an increased activation of these kinases.

**Fig. 21:**
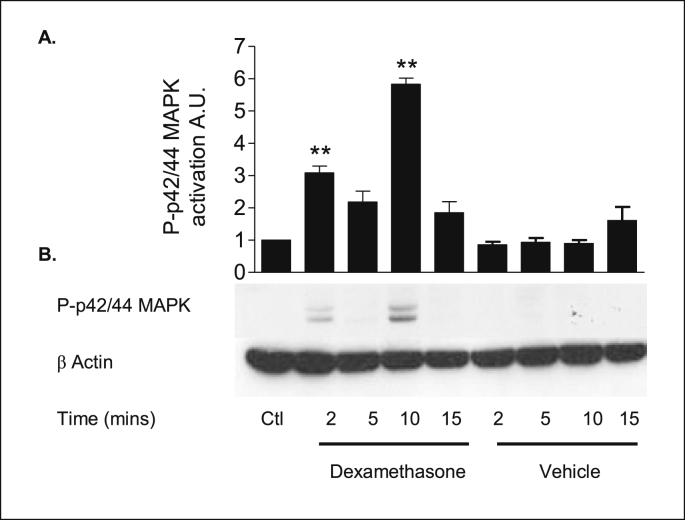
**ERK1/2 MAPK is rapidly activated in response to dexamethasone in 16HBE14o**^**−**^**cells. A:** The graph represents densitometric analysis at specific time points of dexamethasone treatment. Values are given as fold changes in ERK1/2 MAPK activation in 16HBE14o^−^ cell lysates. Values are displayed as mean ± SEM (n = 4). ** Denotes significance (p < 0.001) between control and treated values. Statistical analysis was performed by one-way ANOVA followed by Tukey's multiple comparison tests. **B:** Representative western blot analysis of phospho-ERK1/2 MAPK in cellular extracts of 16HBE14o^−^. By employing antibodies specific to the phosphorylated form of ERK1/2 (Thr 202/Tyr 204), its activation by dexamethasone was monitored by Western blot analysis. Dexamethasone (1 nM) produced a biphasic activation of ERK1/2 that peaked at 2 and 10 minutes.

An antibody specific for phospho-ERK1/2 MAPK was used to determine phosphorylation in response to dexamethasone by western blot analysis. As shown in Fig. 21, dexamethasone (1 nM) produced a biphasic activation of ERK1/2 MAPK. ERK1/2 activation was observed as early as 2 minutes reaching a maximal activation at 10 minutes, thereafter returning to basal levels by 15 minutes. Dexamethasone treatment increased ERK1/2 MAPK phosphorylation levels compared to vehicle controls (Table 14). In 16HBE14o^−^ cells the rapid activation of ERK1/2 MAPK occurs in a time frame consistent with the rapid activation of PKA by dexamethasone.

**Table 14:** Summary of the fold increases in ERK1/2 MAPK activity induced by dexamethasone.

Treatment	Fold increase in ERK1/2 MAPK activity
Lysate control	1 ± 0
Dexamethasone (1 nM, 2 min)	3.08 ± 0.26
Dexamethasone (1 nM, 5 min)	2.17 ± 0.4
Dexamethasone (1 nM, 10 min)	5.83 ± 0.23
Dexamethasone (1 nM, 15 min)	1.6 ± 0.53
Methanol (0.001% v/v, 2 min)	0.85 ± 0.12
Methanol (0.001% v/v, 5 min)	0.93 ± 0.17
Methanol (0.001% v/v, 10 min)	0.89 ± 0.14
Methanol (0.001% v/v, 15 min)	1.6 ± 0.53

### 2.18 Non-genomic response - dexamethasone induces ERK1/2 MAPK activation independent of mRNA translation

**Fig. 22:**
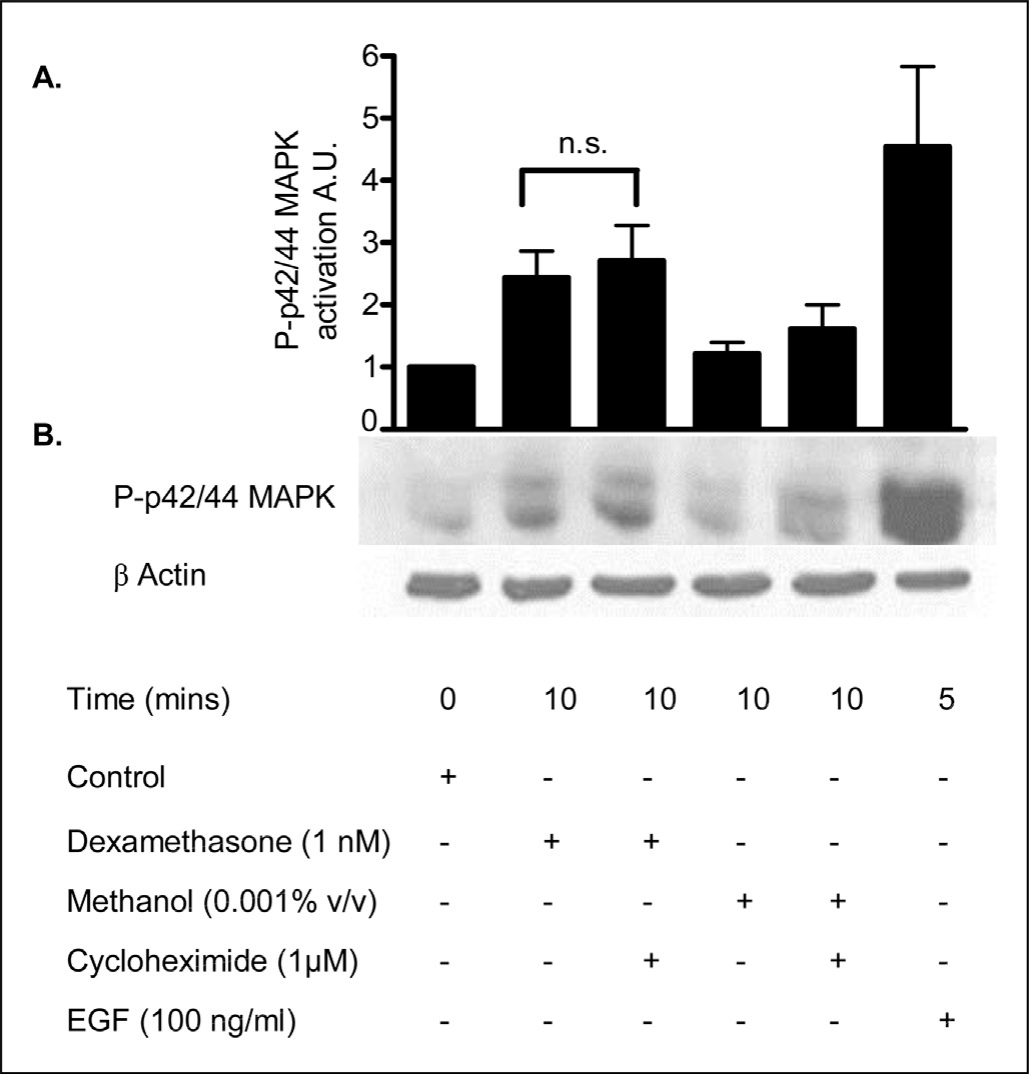
**Dexamethasone induces ERK1/2 MAPK activation independent mRNA translation. A:** The graph represents densitometric analysis at specific time points of dexamethasone treatment. Values are given as fold changes in ERK1/2 activation of 16HBE14o^−^ cell lysates. Values are displayed as mean ± SEM (n = 3); n.s. denotes not significant between dexamethasone and dexamethasone and inhibitor treated values. Statistical analysis was performed by one-way ANOVA followed by Tukey's multiple comparison tests. **B:** Representative Western blot analysis of phospho-ERK1/2 MAPK in cellular extracts of 16HBE14o^−^. The effect of inhibiting translation on the dexamethasone induced MAPK activation was determined by Western blot. Lane 1, lysate control, Lane 2, dexamethasone (1 nM, 5 min), Lane 3, Cycloheximide (prior treatment 1 μM/60 min) and dexamethasone (1 nM, 5 min), Lane 4, vehicle control (methanol, 0.001% v/v, 5 min), Lane 5, vehicle control (methanol 0.001% v/v, 5 min), Lane 6, EGF (100 ng/ml, 10 min).

The time course (5 minutes) of the dexamethasone effect on ERK1/2 MAPK activity suggests that this response does not involve a classical genomic mechanism. In order to verify this hypothesis we tested the dexamethasone (1 nM) response in the presence of cycloheximide, an inhibitor of mRNA translation. As shown in Fig. 22, the increase in ERK1/2 MAPK activity following dexamethasone treatment was not reduced by preincubation with cycloheximide (1 μM) (Table 15). EGF (100 ng/mg) was used as a positive control for MAPK activation. These results indicate a mechanism of dexamethasone-induced activation of ERK1/2 MAPK that is independent of *de novo* protein synthesis.

**Table 15:** Summary of the fold increases in ERK1/2 MAPK activity induced by Dexa.

Treatment	Fold increase in ERK1/2 MAPK activity
Lysate control	1 ± 0
Dexamethasone (1 nM, 10 min)	2.44 ± 0.53
Dexamethasone (1 nM, 10 min) + cycloheximide (1 μM, 60 min prior treatment)	2.17 ± 0.4
Methanol (0.001% v/v, 10 min)	1.21 ± 0.24
Methanol (0.001% v/v, 10 min) + cycloheximide (1 μM)	1.74 ± 0.32
EGF (100 ng/ml, 10 min)	4.54 ± 1.63

### 2.19 Dexamethasone PKA activation is upstream of MAPK activation

**Fig. 23:**
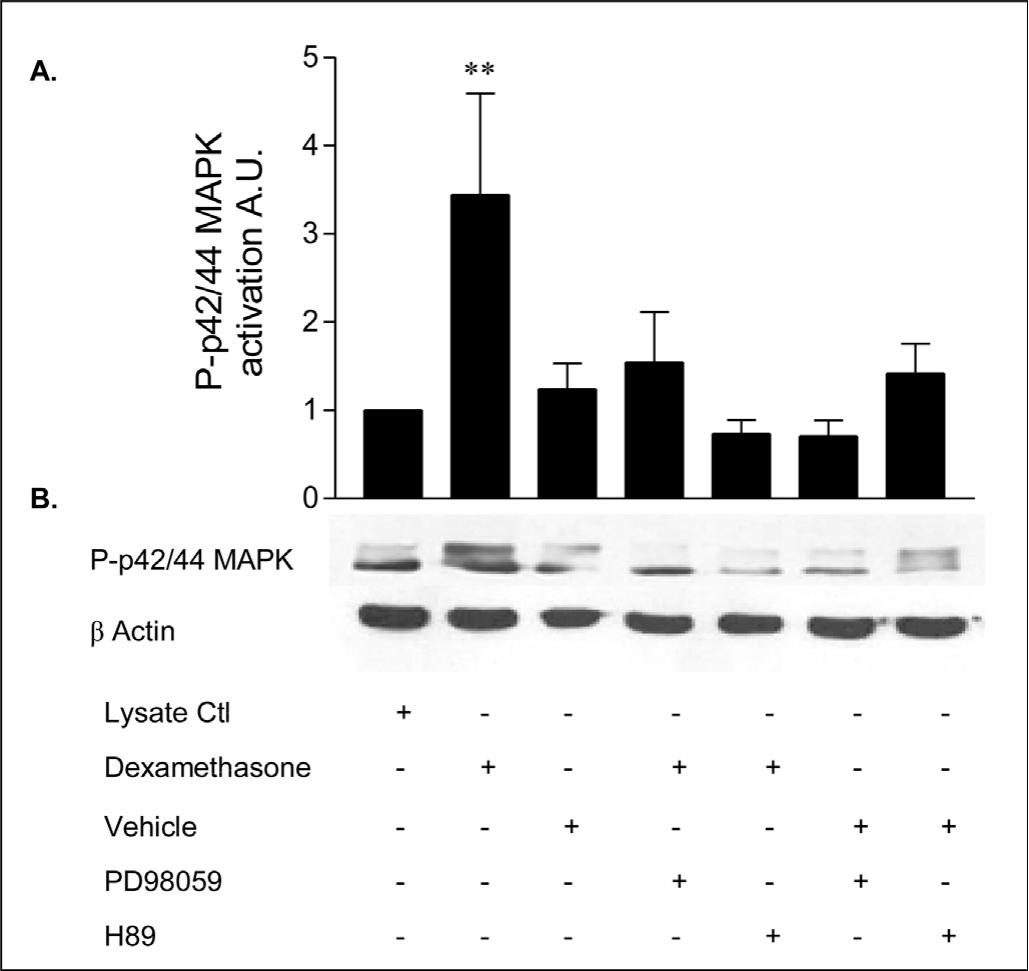
**Effect of the PKA inhibitor, H89 and the MEK1 inhibitor, PD98059 on dexamethasone-induced ERK 1/2-activation. A:** The graph represents densitometric analysis at specific time points of dexamethasone treatment. Values are given as fold changes in ERK1/2 activation of 16HBE14o^−^ cell lysates. Values are displayed as mean ± SEM (n = 3); ** denotes values were significant (p < 0.001) between dexamethasone and dexamethasone and inhibitor treated values. Statistical analysis was performed by one-way ANOVA followed by Tukey's multiple comparison tests. **B:** Representative Western blot analysis of phospho-ERK1/2 MAPK in cellular extracts of 16HBE14o^−^. 16HBE14o^−^ cells were treated with lysate control (Ctl) (*lane 1*), dexamethasone 1nM (*lane 2*), vehicle control (methanol, 0.001%) (*Lane 3*), dexamethasone (1 nM) after pretreatment with PD98059 (25 μM, 20 mins) (*lane 4*), dexamethasone (1 nM) after pretreatment with H89 (10 μM, 20 mins) (*lane 5*). Effect of methanol on inhibitor pre-treatment of PD98059 (*lane 6*) and H89 (*lane 7*).

The potential role of PKA in dexamethasone-induced activation of ERK 1/2 MAPK was examined. As shown in Fig. 23, dexamethasone (1 nM) stimulated ERK1/2 MAPK phosphorylation in 16HBE14o^–^ cells (p < 0.001) (*lane 2*). The rapid activation of MAPK by dexamethasone was significantly inhibited (*p* < 0.001) by the PKA inhibitor H89 (10 μM) (*lane 4*) demonstrating that PKA is activated upstream of ERK1/2 MAPK. As an internal control the MEK 1 inhibitor PD98059 (25 μM) (*lane 5*) inhibited the ERK1/2 MAPK activity. Table 16 shows a summary of fold increases in ERK1/2 MAPK activity.

**Table 16:** Summary of the fold increases in ERK1/2 MAPK activity.

Treatment	Fold increase in ERK1/2 MAPK activity
Lysate control	1 ± 0
Dexamethasone (1 nM, 10 min)	4.0 ± 1.4
Methanol (0.001% v/v, 10 min)	1.4 ± 0.35
Dexamethasone (1 nM, 10 min) + PD98059 (50 μM, 20 min pre-treatment)	1.8 ± 0.7
Dexamethasone (1 nM) + H89 (10 μM, 20 min pre-treatment)	0.8 ± 0.20
PD98059 (50 μM) + methanol (0.001% v/v)	0.73 ± 0.26
H89 (10 μM) + methanol (0.001% v/v)	1.65 ± 0.36

### 2.20 PKC activation is upstream of PKA

The data indicate that dexamethasone induced MAP kinase activation is dependent on upstream PKA activity. What dexamethasone modulated kinase, if any, is upstream of PKA ?

**Fig. 24:**
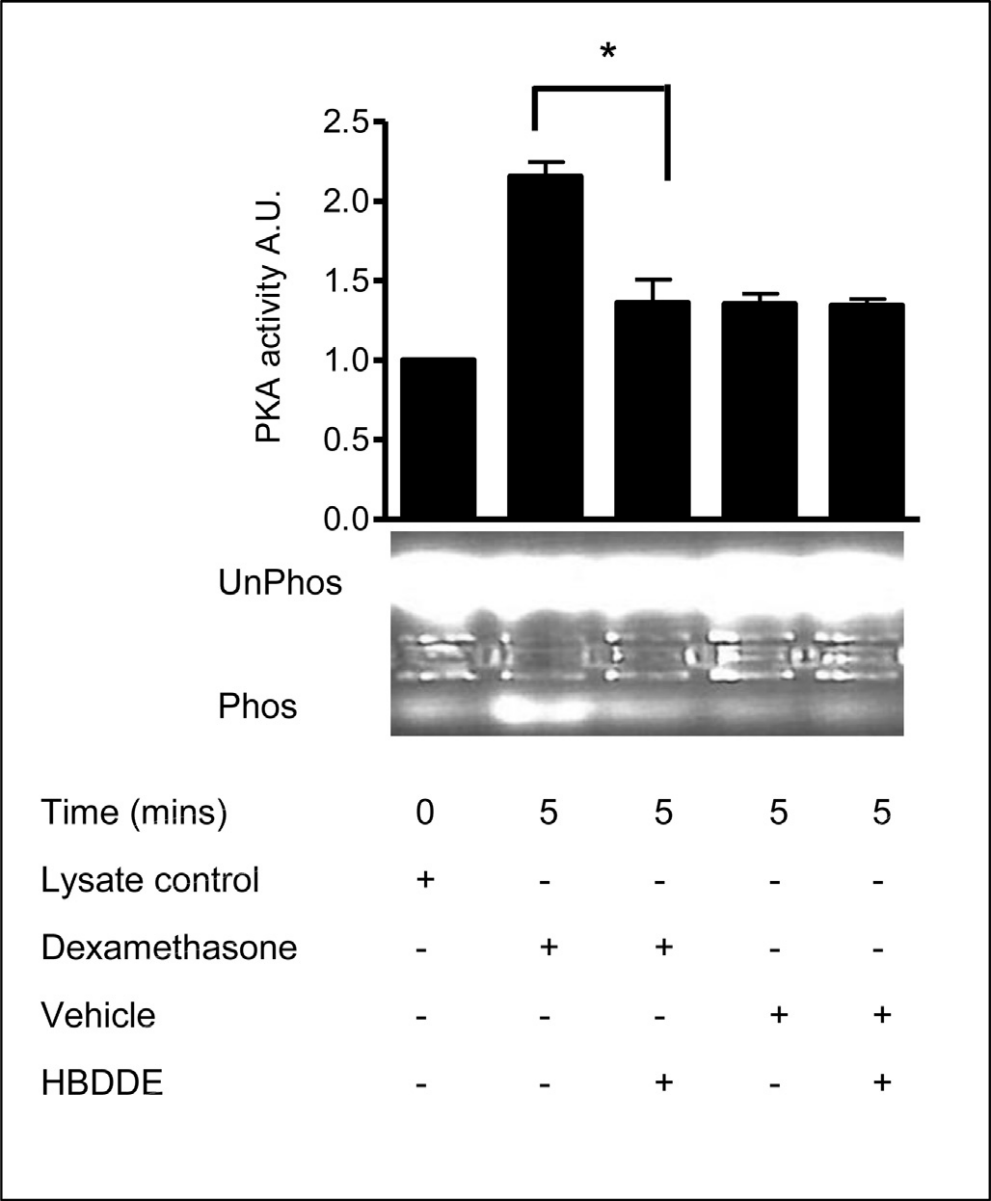
**PKA activated in response to dexamethasone and is downstream of PKC**α**.** The graph represents densitometric analysis of treatments. Values are given as fold changes in PKA activation measured as phosphorylation of the F-Kemptide PepTag for 16HBE14o^−^ cell lysates. Values are displayed as mean ± SEM (n = 3), * denotes (p > 0.01) between dexamethasone and dexamethasone plus PKC α inhibitor. Statistical analysis was performed by one-way ANOVA followed by Tukey's multiple comparison tests. UV-illuminated agarose gel of the products of reactions run with F-Kemptide and 16HBE14o^−^ homogenate. Lane 1, lysate control; Lane 2, dexamethasone (1 nM, 5 min); Lane 3, HBDDE (prior treatment 100 μM/40 min) and dexamethasone (1 nM, 5 min) Lane 4, vehicle control (methanol, 0.001% v/v, 5 min), Lane 5, methanol 0.001% v/v, 5 min and vehicle control (DMSO 0.1% v/v) for HBDDE.

In order to determine whether PKC activation was involved in the dexamethasone induced activation of PKA, the effects of the PKCα inhibitor 2,2′,3,3′,4,4′-Hexahydroxy-1,1′-biphenyl-6,6′-dimethanol Dimethyl Ether (HBDDE), a selective inhibitor of PKCα and PKCγ (IC_50_ concentration: PKCα, 43 μM and PKCγ 50 μM) was examined. PKCγ was not expressed in the human airway epithelial cells (it is exclusively expressed in brain and spinal tissue). Dexamethasone induced PKA activation was calculated as fold increases (arbitrary units, A.U.). Serum starved cells were treated with HBDDE (100 μM) for 30 mins before dexamethasone treatment (1 nM). Vehicle and HBDDE on its own had no effect on basal PKA activity. However, pretreatment with the PKCα inhibitor HBDDE (100 μM) prevented dexamethasone induced PKA activation (Fig. 24, Table 17). This result shows that PKA activated in response to dexamethasone is downstream of PKCα.

**Table 17:** Summary of the fold increases in PKA activity.

Treatment	Fold increase in PKA activity
Lysate control	1 ± 0
Dexamethasone (1nM, 5 min)	2.15 ± 0.12
Dexamethasone (1 nM, 5 min) + HBDDE (100 μM, 30 min prior treatment)	1.36 ± 0.18
Methanol (0.001% v/v, 5 min)	1.35 ± 0.08
Methanol (0.001% v/v, 5 min) + HBDDE (100 μM, 30 min prior treatment)	1.34 ± 0.05

### 2.21 Effect of dexamethasone on PKD1 activation in 16HBE14o^−^ cells

**Fig. 25:**
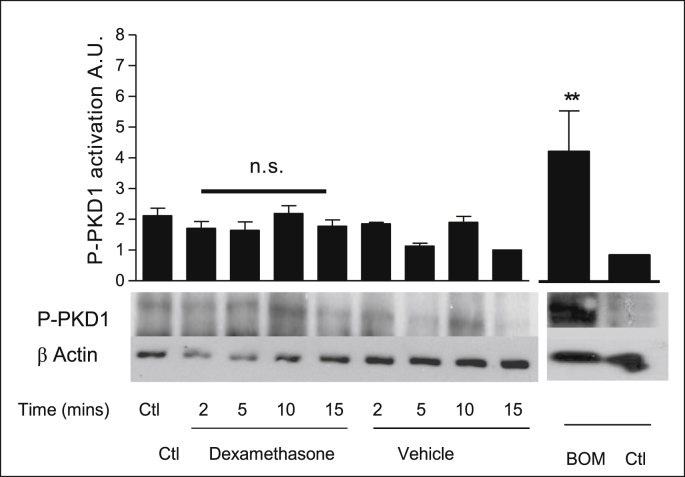
**Dexamethasone does not activate PKD1 in 16HBE14o**^**−**^**cells.** Representative western blot analysis of phospho-PKD1 in cellular extracts of 16HBE14o^−^. By employing antibodies specific to the phosphorylated form of PKD1 (Ser 916), the activation by dexamethasone was monitored by Western blot analysis. β-actin (42 kDa) was used as an internal control to estimate protein loading. The graph represents densitometric analysis at specific time points of dexamethasone treatment. Bombesin (BOM) was used as a positive control for PKD1 activity. Values are given as fold changes in PKCε activation of 16HBE14o^−^ cell lysates. Values are displayed as mean ± SEM (n = 3); n.s. denotes no significance between control and treated values. Statistical analysis was performed by one-way ANOVA followed by Tukey's multiple comparison tests.

Protein kinase D is a potential downstream target of cPKC and nPKCs. To determine whether dexamethasone stimulated activation of PKD1 in 16HBE14o^−^ cells, serum starved cells were treated with dexamethasone (1 nM) and PKD1 activity was assessed by probing with an antibody specific to phosphorylation to Ser916. Residue Ser916 is a site of autophosphorylation in the PKD1 structure that occurs subsequent to phosphorylation at Ser744/748 by PKC. Phosphorylation at Ser916 is therefore indicative of PKD activation. Treatment of 16HBE14o^−^ cells with dexamethasone had no significant effect on PKD1 phosphorylation levels (Fig. 25, Table 18). Bombesin was used as a positive control as it is known to activate PKD1.

**Table 18:** Summary of the fold increases in PKD activity.

Treatment	PKD activation (phosphorylation at Ser 729)
Lysate control	2.27 ± 0.26
Dexamethasone (1nM, 2 min)	1.7 ± 0.27
Dexamethasone (1nM, 5 min)	1.64 ± 0.32
Dexamethasone (1nM, 10 min)	2.19 ± 0.32
Dexamethasone (1nM, 15 min)	1.78 ± 0.26
Methanol (0.001% v/v, 2 min)	1.96 ± 0.06
Methanol (0.001% v/v, 5 min)	1.13 ± 0.13
Methanol (0.001% v/v, 10 min)	2.11 ± 0.30
Methanol (0.001% v/v, 15 min)	1.92 ± 1.16
Bombesin	4.13 ± 0.31
Control	1.4 ± 0.29
